# MDCT Diagnosis and Staging of Xanthogranulomatous Pyelonephritis

**DOI:** 10.3390/diagnostics13071340

**Published:** 2023-04-04

**Authors:** Stefania Tamburrini, Rosita Comune, Giulia Lassandro, Filomena Pezzullo, Carlo Liguori, Valeria Fiorini, Stefano Giusto Picchi, Marina Lugarà, Dario Del Biondo, Salvatore Masala, Fabio Tamburro, Mariano Scaglione

**Affiliations:** 1 Department of Radiology, Ospedale del Mare-ASL NA1 Centro, Via Enrico Russo 11, 80147 Naples, Italy; giulia.lassandro@gmail.com (G.L.);; 2 Department of Precision Medicine, Section of Radiology and Radiotherapy, University of Campania “Luigi Vanvitelli”, 80128 Naples, Italy; 3 Department of Internal Medicine, Ospedale del Mare-ASL NA1 Centro, Via Enrico Russo 11, 80147 Naples, Italy; marinalugara82@gmail.com; 4 Department of Urology, Ospedale del Mare-ASL NA1 Centro, Via Enrico Russo 11, 80147 Naples, Italy; 5 Department of Medical, Surgical and Experimental Sciences, University of Sassari, 07100 Sassari, Italy; 6 James Cook University Hospital, Middlesbrough TS4 3BW, UK

**Keywords:** xanthogranulomatous pyelonephritis, complicated pyelonephritis, renal stone disease, nephrolithiasis, infection, inflammation, kidney diseases, MDCT, multidetector computed tomography, benign nephrectomies

## Abstract

**Background**: Benign nephrectomy to treat patients with renal inflammatory disease in cases of severe urinary infection represents a diagnostic and management challenge because of significant inflammatory, fibrotic, and infectious components. Among renal inflammatory diseases, fistulization and invasiveness to adjacent structures are some of the hallmarks of xanthogranulomatous pyelonephritis (XGP). The aims of this study were as follows 1. to retrospectively determine key demographic and clinical features of XGP among benign nephrectomies; 2. to assess the CT preoperative diagnostic accuracy; and 3. to define the imaging characteristics of the CT stage. **Material and Methods**: A retrospective review of clinical, laboratory, and radiological features and operative methods of patients who underwent benign nephrectomy with histologically proven XGP was performed. **Results**: XPG was diagnosed in 18 patients over a 4-year (2018–2022) period. XGP represented 43.90% among benign nephrectomies. The mean age of the patients was 63 years, and the sex prevalence was higher in women (72.22%). Symptoms were vague and not specifically referrable to urinary tract disorders and unilateral (100%), with the left kidney affected in 61.11% of cases. Staghorn calculi and stone disease were the most common underlying cause (72.22%). All patients underwent CT. The preoperative CT imaging accuracy for renal inflammatory disease was 94.44% and indeterminate in 5.56%. A suspected diagnosis of XGP was formulated in 66.67% (12/18; 2 stage II/10 stage III), meanwhile, in 33.33% (6 patients with stage I), a non-specific diagnosis of renal inflammatory disease was formulated. CT was reported according to the Malek and Elder classification and staged in the stage I nephric form (33.33%), stage II perinephric form (11.11%), stage III paranephric form (55.56%). **Conclusions**: The CT diagnostic accuracy for kidney inflammatory disease was extremely high, whereas the suspected diagnosis of XGP was formulated preoperatively in only 66.67% of high-stage disease, where the hallmarks of invasiveness and fistulization of the pathology increased the diagnostic confidence.

## 1. Introduction

The prevalence of upper urinary tract stones is approximately 8% of the population, and its incidence has increased worldwide [1]. The treatment is focused on preserving renal function and eradicating kidney stones, performing minimally invasive techniques, such as percutaneous nephrolithotomy, shock wave lithotripsy, and ureteroscopy [2]. Despite new advances in treatment, there are still some patients requiring simple nephrectomy because of poor renal function with recurrent infections, pain, abscess, fistulization, and suspected malignant transformation [3]. Fistulization is one of the hallmarks of xanthogranulomatous pyelonephritis (XGP). XGP accounts for 0.6–1% of renal inflammatory diseases, and it is characterized by suppurative granulomatous inflammation and the progressive destruction of the renal parenchyma secondary to chronic urinary obstruction caused by renal calculi, ureteropelvic junction obstruction, vesicoureteral reflux, chronic interstitial nephritis, or external compression from adjacent masses [4,5,6,7,8,9,10]. In XGP, the renal tissue is replaced by a cellular infiltrate of lipid-laden (foamy) mononuclear macrophages; this destructive process may be profound and can infiltrate surrounding tissues and viscera [11,12]. The precise etiology of XGP is not fully understood, although it is strongly associated with urinary infections, chronic urinary obstruction, and an altered immune response. Other etiological factors have been postulated to play a role, including obesity, immunosuppression, dyslipidemia, and diabetes mellitus [13]. Recently, infliximab and other anti-TNF biologics have been reported to increase the risk of XGP in patients with predisposing factors [14]. Diffuse XPG is more frequent in middle-aged women, after the thirties and especially in the fifties, although pediatric cases have been reported [5,15,16]. The clinical presentation can be insidious, determining the delayed diagnosis. Common symptoms are general malaise, dysuria, flank pain (non-colicky), hematuria, fever, chills, unintentional weight loss, and loss of appetite; symptoms have usually been present for some time, longer than 6 months in 42% of patients [17,18]. In some cases, patients can present only with signs and symptoms related to superimposed complications, such as shortness of breath as a result of empyema caused by intrathoracic communication or discharging sinus from the flank wall as a result of renocutaneous fistulation [13,19,20]. A delay in diagnosis can contribute to epithelium dedifferentiation and dysplasia and favor cancer development, increasing the chance of its occurrence more than twice [21,22]. XGP is difficult to distinguish from pyonephrosis, renal tuberculosis, and renal cell carcinoma, as well as tuberculosis, renal abscess, angiomyolipoma, extrapulmonary sarcoidosis, and actinomycosis, by clinical symptoms, physical examination, and image findings; thus, generally, it is confirmed by pathological examination after nephrectomy [4,13,23]. XGP may be a life-threatening condition and generally, the best management is radical surgery, as conservative measures with antibiotics fail to treat the underlying pathological process [23,24,25]. In this scenario, patient demographics and clinical and imaging findings can aid in the early identification and treatment of patients at risk. Three different types of XGP are described: the diffuse form accounting for 90% of cases, and the segmental and focal form, which account together for 13% of cases; XGP can also affect renal allografts [13]. The diffuse variant involves the entire kidney, and a three-level grading system was formulated by Malek and Elder [26]. In the segmental form, the disease is limited to the renal cortex and there is no involvement of the renal pelvis [14,27]. The aims of this study were to as follows: 1. retrospectively determine key demographic and clinical features of XGP among benign nephrectomies; 2. to assess the CT preoperative diagnostic accuracy; and 3. to define the imaging characteristic of the CT stage.

## 2. Materials and Methods

The local Institutional review board approved the study. The Ethical Committee approval was obtained (2022030). All participants of this study provided written informed consent for collection of data in order to use them for scientific purposes. 

### 2.1. Patient Population and Study Design

We retrospectively retrieved, from surgical and pathological reports, all consecutive patients, >18-years-jold, undergoing benign nephrectomy between September 2018 and July 2022 at our institution. Recorded variables included age, sex, and technique of surgery. Key clinical, laboratory, and histologic features were analyzed. Pre-operative radiological diagnosis and staging were compared to the final histological diagnosis. Computed tomography was obtained preoperatively for all patients. The final diagnosis was defined histologically as XGP with the presence of a focal, segmentary, or diffuse process characterized by the presence of foam-laden macrophages within a chronic inflammatory background [28].

### 2.2. CT Protocol

**Figure 1:**
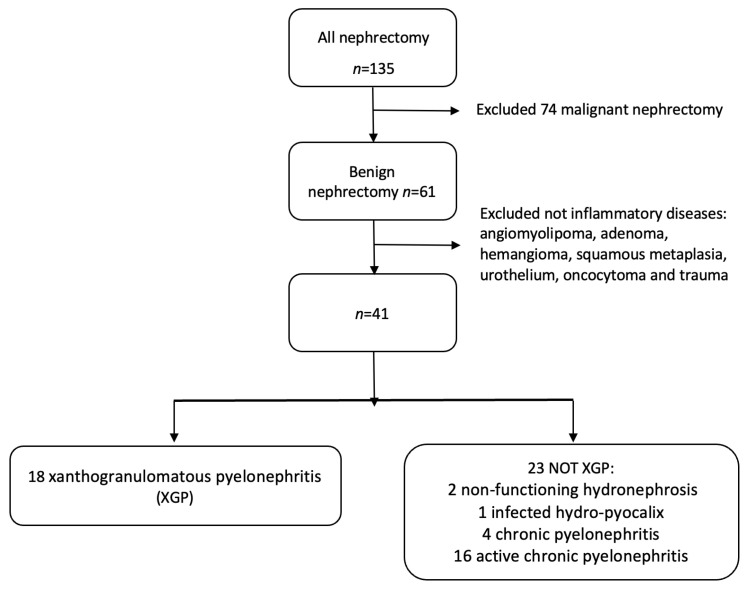
Flow chart of patients included in the study. Among them, 41 cases of inflammatory renal diseases were diagnosed: 2 non-functioning hydronephrosis, 1 infected hydro-pyocalix, 4 chronic pyelonephritis, 16 active chronic pyelonephritis, and 18 XGP.

**Figure 2:**
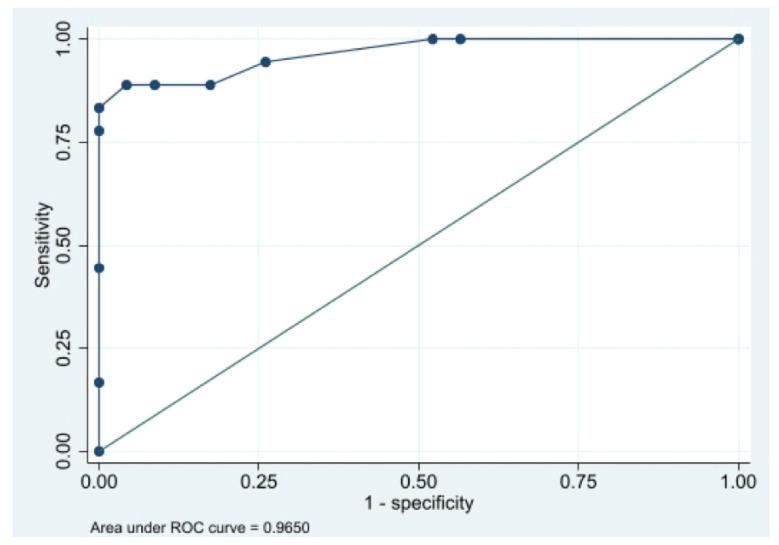
ROC curve of prediction model.

**Figure 3:**
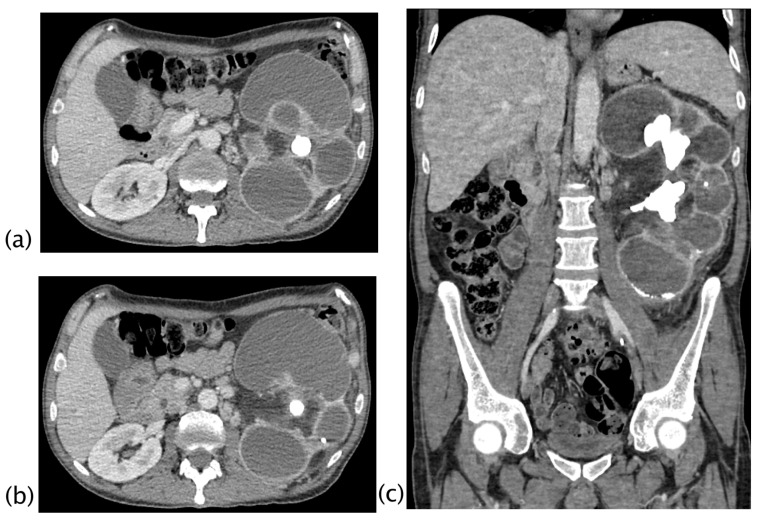
Xanthogranulomatous pyelonephritis Stage I—nephritic form. CT with contrast agent: axial (**a**,**b**) and coronal MPR reconstruction (**c**). The left kidney is increased in volume with a characteristic “contracted” appearance of the renal pelvis. The renal parenchyma is extremely thinned and characterized by the presence of multiple hypodense areas representing both intraparenchymal collections of pus and dilated calyces. The wall of the pyelocaliceal structures is thickened and hyperemic due to granulation tissue and parenchymal compression caused by abscess cavities. Staghorn lithiasis is present. Perirenal fat is thickened but no peri- or pararenal collections are present. No fistulas or abscesses are evident. The characteristic “bear paw sign” and contracted appearance of the pelvis allowed for the diagnosis of XGP, confirmed on histologic examination. The “bear paw sign” was appreciated on the CT scan in 66.67% of patients.

**Figure 4:**
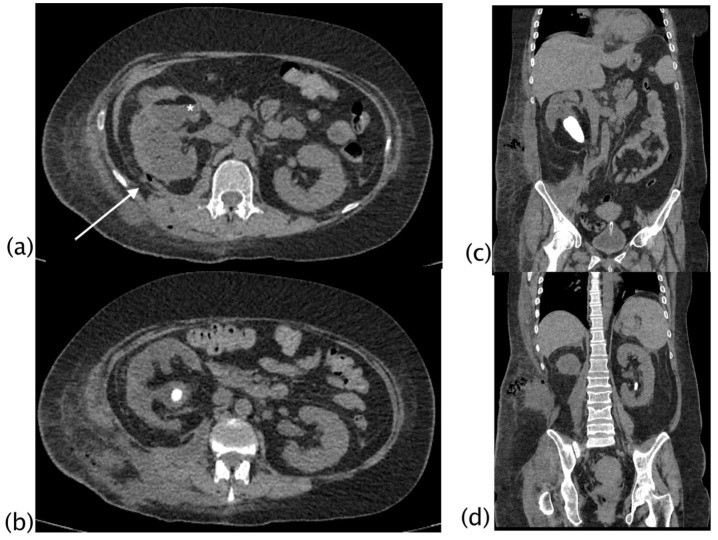
Xanthogranulomatous pyelonephritis Stage III—pararenal form. Non-enhanced CT examination: axial (**a**,**b**) and coronal MPR reconstruction (**c**,**d**). The right kidney is increased in volume with reduced parenchymal representation and a “contracted” appearance of the renal pelvis. The renal parenchyma is extremely thinned and characterized by the presence of multiple hypodense areas that cannot be better characterized without contrast agent. (**a**) Within the fluid collection at the anterior middle third of the kidney, a “layer” characterized by fluid (white asterisk) and adipose density (above white asterisk) can be noted, named “xanthoma” and characteristic of the disease. Staghorn lithiasis is present. No significant thickening of the perirenal fat tissue is noted. On the posterior profile of the kidney, a fistulous via with hydro-gas content is visible ((**a**), white arrow; (**b**)), which carries posteriorly into the psoas and dorsal muscles with fluid collection in the context of the muscle bundles. The fistulous via terminates in the subcutaneous flank soft tissues where fluid collection with gas is present ((**d**), MPR coronal).

MDCT exams were performed using the Aquilion Prime—160 channels system (Toshiba Medical Systems, Otawara City, Japan). At our institution, a volumetric multi-phase CT examination is acquired for renal pathologies. A non-contrast scan is performed in order to identify calculi and intralesional fat and to determine the homogeneity and UH values of focal lesions. After that, intravenous contrast (1.0–1.5 mL/kg) is injected at 3–5 mL/s (possibly higher flows, depending on the vein access), followed by 60 mL of a saline bolus injection. The corticomedullary phase is obtained with a delay of 25 s (threshold of 120 HU within the abdominal aorta before renal artery origins). The nephrogenic phase is obtained with a delay of 90 s, to ensure optimal enhancement of the renal parenchyma and to underline the enhancement and washout of renal lesions. A delayed excretory phase is obtained after at least 8 min (400–500 s). In cases of suspected urothelial carcinoma or extra-renal extension of the disease (i.e., urinoma in the retroperitoneal spaces, or abscesses in communication with the calico-pelvic system), a delayed phase is acquired after at least 20–30 min. Depending on the localization of the suspected urothelial carcinoma, the delayed phase can be acquired in the prone position. CT exams were retrospectively reviewed in a consensus reading by two radiologists with experience in abdominal radiology (FP, MS) and a radiologist with experience in urological radiology (ST). CT imaging findings of lithiasis, staghorn calculi, hydro- and pyonephrosis, the presence of blood or air in the collecting system, a “bear paw sign”, xanthomas, fat stranding, abscess, pleural effusion, fistulization, and adherence to surrounding structures (Figure 1, Figure 2, Figure 3 and Figure 4) were recorded. 

### 2.3. Statistical Analysis

Descriptive statistics were calculated for included patients. Categorical variables were reported as counts and percentages. Continuous variables were expressed as the mean ± standard deviation (SD) if normally distributed or as the median and interquartile range (IQR) if non-normally distributed. For the comparison between XGP and not-XGP inflammatory diseases, categorical variables were compared using a χ^2^ test or Fisher’s exact test as appropriate, whereas continuous variables were compared using a Student’s t-test or Mann–Whitney U test depending on whether they were normally distributed or not. To investigate risk factors for XGP, univariable and multivariable logistic regression analyses were performed. Odds ratios (ORs) and their corresponding 95% confidence intervals (95% CIs) were estimated. The covariates to be included in the multivariable logistic regression models were selected through a backward stepwise selection strategy (*p*-value for inclusion ≤ 0.1, *p*-value for exclusion > 0.2). Variables were primarily entered according to clinically and statistical relevance and lack of collinearity. Age was entered as a binary variable according to Youden’s criterion while, the cut-offs for the other variables were based on the clinical reference. 

## 3. Results

A total of 135 patients, who underwent nephrectomy, were included in the study. Of these, 74 malignant nephrectomies were excluded from the analysis. Fifty-five patients were admitted to the Urology Department for suspected renal carcinoma, 14 were admitted through the Emergency Department, and five were hospitalized in other departments with an incidental diagnosis of renal carcinoma. Forty-seven patients had a focal renal lesion (34 clear cell carcinoma, 2 cortical renal cell carcinoma, 10 papillary renal cell carcinoma, 1 fibrous solitary tumor). Among them, three patients presented signs of chronic pyelonephritis (one clear cell carcinoma, one papillary renal cell carcinoma, one fibrous solitary tumor). The preoperative suspected diagnosis was of focal renal carcinoma. Two patients underwent nephrectomy for metastatic disease (gastric adenocarcinoma, pancreatic adenocarcinoma).

Twenty-five patients had urothelial cell carcinoma; five patients with urothelial carcinoma had hematuria and signs of obstruction and chronic pyelonephritis with mild hydronephrosis in the absence of calculi. The obstruction was represented by a solid tumor within the calico-pelvic system. In none of them, a preoperative suspected diagnosis of chronic inflammatory renal disease or XGP was formulated. Lipid-foam macrophages were not found at histology in none of the patients with malignancy. 

A total of 61 patients underwent benign nephrectomy (52 total nephrectomy, 9 partial nephrectomy). A flowchart of included patients is shown in Figure 1. Of the 41 patients included in the study, 17 were admitted through the emergency department for acute symptoms, 22 were scheduled for nephrectomy and hospitalized in the Urology Department, and 2 patients were hospitalized for severe weight loss (1/41) and pneumonitis (1/41). Of the 18 patients with XGP, 9/18 were admitted through the ER, 8 were admitted to the Urology Department, and 1 was hospitalized for a persistent cough. Demographic, clinical, and laboratory values and the prevalence of CT findings in XGP were compared to those of the other renal inflammatory diseases (Table 1). The incidence of XGP between renal inflammatory disease was 43.9%. The mean age of the patients was 60 years (SD = 17); there were 26 women (63.4%) and 15 men (36.6%). The most common symptoms were flank or abdominal pain (28/41), fever (10/41), hematuria (9/41), and a palpable mass (2/41). More than 70% of XGP patients suffer from diabetes, and XGP patients also reported a lower median than other patients in hemoglobin and cholinesterase and a higher median in leucocyte values. All patients underwent CT, 39/41 with intravenous contrast and 2/41 without contrast for renal impairment; among XGP, 16 patients underwent CT with intravenous contrast, with 2 patients without contrast. CT imaging showed large differences, which were statistically significant, between XGP patients and the non-XGP group for the presence of staghorn calculi, a bear paw sign, pyonephrosis, air in the collecting system, fat stranding, renal abscess, pararenal abscess, perirenal abscess, spleen abscess, psoas abscess, pleural effusion, and lung atelectasis/disventilation. More than 60% of XGP patients showed, at CT imaging, a bear paw sign, hydro- and pyo-nephrosis, fat stranding, renal and perirenal abscess, and lung atelectasis/disventilation (Table 1). The underlying cause of obstruction was staghorn calculus in 9/18, and overall, 15/18 patients had renal stone disease. Three patients without a clear obstruction had a congenital pelviureteric junction. The classic CT appearance of XGP referred to as the ‘‘bear paw sign’’, due to the loss of the normal renal outline with a paradoxical contracted pelvis in contrast to the multiloculated appearance of dilated calyces [29], was appreciated in 13/18 cases (72.2%). In only one XGP patient, a demonstrable focal fat deposit representing the pathologically distinguishing feature of xanthomas was detected (5.6%) (Table 1). All cases of inflammatory benign nephrectomies were unilateral, with the left kidney involved in 22 cases (53.7%) and the right kidney in 19 cases (46.3%). In XPG, the left kidney was involved in 11/18 patients (61.1%), whereas the right kidney was affected in 7/18 cases (38.9%). Before surgery, drainage of the infected and obstructed calico-pelvic system was performed in six patients with XGP and in five patients with chronic pyelonephritis. At CT, the presence of blood in the collecting system was strongly related to stent insertion (*p* < 0.001) and it was not significant for the diagnosis of XGP (*p* = 0.267); oppositely, the presence of air in the collecting system was not related to stent insertion (*p* = 0.069), and it appeared to be significant for the diagnosis of XGP (*p* = 0.031). The characteristic “bear paw sign”, the contracted appearance of the pelvis, xanthoma, and fistula allowed for a confident diagnosis of XGP. The identification of xanthoma was appreciated on CT in 5.56% of patients.

Nephrectomy was accomplished due to pain in excluded renal units or severe urinary infection and invasiveness or fistulization to adjacent organs. CT was discussed with urologists to define surgical planning and the operative treatment: 29 patients underwent laparoscopy, 12 were operated by a laparoscopic approach, and 9 by a robotic surgical approach. Among patients with XGP, 9/18 patients underwent laparotomy, 8 patients underwent laparoscopy, and only one patient was operated by a robotic surgical approach. Univariable analysis of risk factors is reported in Table 2. Age more than 55 years, diabetes, high value of leukocytes, low value of cholinesterase, presence at CT of staghorn stone, pyonephrosis, air in the collecting system, fat stranding, renal, perirenal, pararenal, and psoas abscess, pleural effusion, and lung atelectasis resulted in the more clinically significant factors investigated for their ability to predict the diagnosis of XGP. Odds ratios for these risk factors ranged from 4.0 (for diabetes) to 28.8 (for pyonephrosis), and they were used for the multivariable analysis. Based on results obtained from univariable analysis, we construct a biomarker-based risk prediction model. Four risk factors, including age more than 55 years, presence at CT findings of staghorn stone, pyonephrosis, and pleural effusion were used for the XGP risk prediction model. This model integrates multiple risk factors to individuate the presence of XGP (Table 3). The prediction model showed a sensitivity of 88.9%, specificity of 91.3%, accuracy of 90.2%, and AUC of 0.97 (Figure 2). 

**Figure 5:**
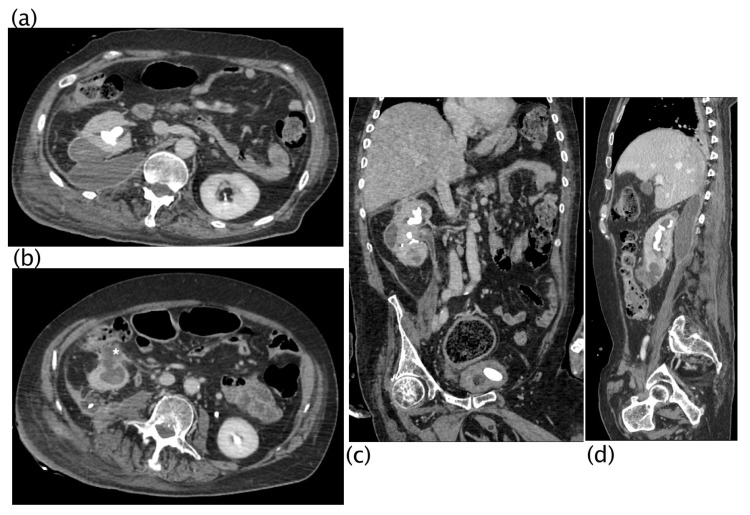
Xanthogranulomatous pyelonephritis Stage III—pararenal form CT with contrast agent: axial (**a**,**b**), coronal (**c**), and sagittal (**d**) MPR reconstruction. In the right kidney, staghorn lithiasis is present; gross vesical lithiasis is also visible. The walls of the ureter are thickened and hyperemic. The CT was performed after nephrostomy placement, so significant calico-pyelic dilatation is not significant. Peri- and pararenal collections are evident, particularly the inferior polar collection (**c**) that passes the perirenal fascia. Posteriorly (**b**), the inflammatory process passes and infiltrates the psoas muscle. The staghorn lithiasis, the coexistence of calico-pyelic dilatation and fluid collections, and especially, the high invasiveness of the pathology, allowed, with high diagnostic confidence, to make the diagnosis of XGP.

**Figure 6:**
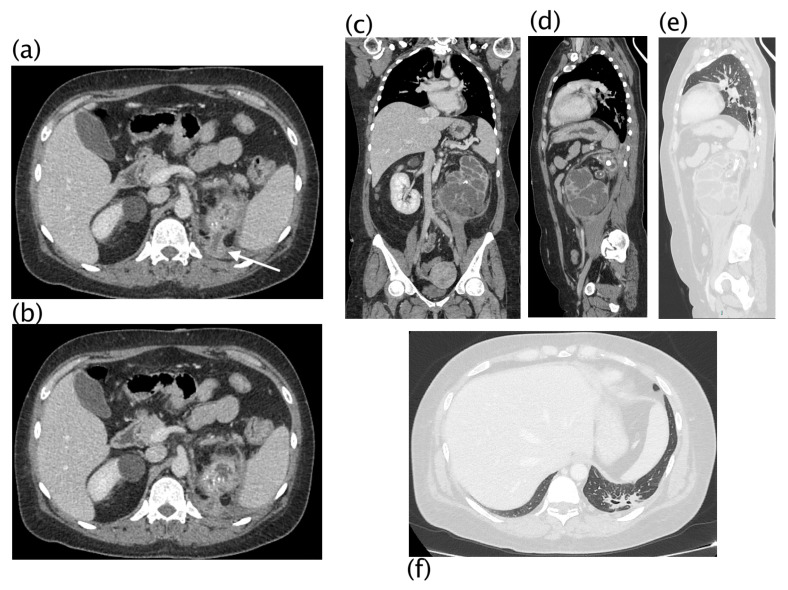
Xanthogranulomatous pyelonephritis Stage III—pararenal form CT with contrast agent: axial (**a**,**b**,**e**), coronal (**c**), and sagittal (**d**) MPR reconstructions with lung window (**e**,**f**). Patient admitted for persistent cough and left basal opacity on chest X-ray examination. On CT examination, morpho-structural alteration of the kidney with gross intraparenchymal collections alternating with dilated calyces is present. An upper polar fistulous via (**a**,**b**) is well evident (white arrow) that continues into the lung in direct continuity with a bronchus, more evident in sagittal reconstructions. Recognition of the fistulous via on CT was visible in 22.22% of patients.

In 2/18 (11.11%) patients, a focus of low-grade papillary carcinoma was found at the histological examination. XGP was suspected preoperatively as a possible diagnosis in 66.7% of the cases (12/18; 2 patients stage II and 10 patients stage III), and in the other 33.3% (6 patients with stage I), a non-specific diagnosis of renal inflammatory disease was formulated. The overall preoperative CT imaging accuracy for renal inflammatory disease was 94.4% (40/41) and was indeterminate in 5.6% (1/41). In one case, a focal lesion with undefined margins and corticomedullary extension was reported as indeterminate. The patient underwent PET-CT examination that showed the absence of FDG uptake and a nephron-sparing surgery was performed; the final histologic diagnosis was a focal form of XPG. CT findings in the three different stages of XGP were reported; fistulization was a typical finding of XGP in stage III, although the sample size did not allow for inferential conclusions (Table 4 and Figure 3, Figure 4, Figure 5 and Figure 6).

## 4. Discussion

XGP was first described by Schlagenhaufer in 1916; however, the current description was not applied until Osterlind in 1944. XGP is an aggressive variant of chronic pyelonephritis resulting in a non-functioning kidney [10,30]. XGP accounts for 0.6–1% of renal inflammatory diseases; however, our rate of XGP was 43.9% among benign nephrectomies for renal inflammatory disease, and it was much greater than that previously reported. We have presented one of the largest series of patients in a four-year period, according to other authors that had previously noted a higher prevalence [28,31,32]. Several contributing and interrelated factors may lead to this; first of all, the high incidence in our population may be explained because the study was carried out during the COVID-19 pandemic. In this period, many hospitals in our district were converted to Covid Centers, and our institution represented one of the few referral centers for all other diseases. Secondarily, in our region, the urological departments are not present in all the hospitals. Thirdly, we selected only patients who underwent nephrectomy, which represents the treatment of choice for XGP in adults. 

The diagnosis of XGP is challenging, and it is often confused with renal cell carcinoma [10], especially in its focal or segmental forms. On the other hand, the invasiveness of XGP to adjacent organs, such as the liver, spleen, duodenum, pancreas, and great vessels, can be interpreted as a sign of malignancy [33]. Surgery is the treatment of choice in adult patients, being linked to an excellent prognosis and a decrease in morbidity and mortality rates [34,35]. Conservative treatment with antibiotics is usually reserved for the pediatric population as first-line therapy, especially in focal forms; meanwhile, in the case of large obstructive calculi, or coraliform, as in the appearance of pyonephrosis on lithiasis, surgery may be the only option even for pediatric ages [36]. 

The pathomechanism of XGP is not fully understood. Many factors are implicated: chronic obstructive disease with ongoing ineffectively treated chronic urinary infections, an altered immune response, and disorders of lipid metabolism [30,36]. Calculi serve as a nidus for infection; organisms most commonly associated with XGP are *Escherichia coli*, *Proteus mirabilis*, *Pseudomonas*, *Enterococcus faecalis*, *and Klebsiella* [28]. Renal parenchyma and peri-renal tissue are destructed and replaced by granulomatous tissue containing lipid-laden macrophages that appear yellow in the pathological section [16,37,38,39]

The demographic distribution in our study confirmed the higher incidence in women, a median age of 63 years, and left kidney prevalence (61.1%) [23,25]. In line with available literature, no significative laboratory finding alterations were found [25,28,40,41]; however, in our patients, C-reactive protein, blood urea nitrogen, and liver function were often mildly elevated. MDCT with intravenous contrast represents the imaging of choice for these patients. MDCT allows for the identification of renal parenchyma replacement by multiple fluid collections with thick and hyperemic layers, which represent the dilated and infected collecting system. MDCT is also accurate in identifying calculous disease and urinary obstruction, and in staging the disease, determining the invasion of adjacent organs and structures [4]. Two CT signs have always been considered highly specific for the diagnosis of XGP, the “bear paw” sign and the presence of xanthoma. The “bear paw “sign is a highly specific CT finding for the diagnosis of XGP; in our study, the “bear paw” sign was found in 72.2% of patients with XGP, and it was never detected in the control group represented by non-XGP renal inflammatory disease [9,10,11,13,18,23,42,43,44]. Xanthoma was found only in 5.6%, and although it is considered pathognomonic for the diagnosis, it appeared to be extremely rare in our study. 

In our retrospective study, the CT overall diagnostic accuracy for the diagnosis of XGP was of 66.7%; according to our results, the correct identification of the “bear paw” sign could increase the CT diagnostic accuracy in discriminating XGP from other renal inflammatory diseases, allowing for a diagnosis in earlier stages and avoiding late complications. 

The surgical approach (laparotomy versus laparoscopic) at our institution reflected what was previously published by Danilovic et al. [2]: open radical nephrectomy remains the most common, and laparoscopic and partial nephrectomy are feasible in well-selected patients. Surgical planning should be discussed by urologists and radiologists based on CT imaging to define the safest approach; in cases where CT imaging describes para and perirenal abscess, a laparotomy approach should be directly performed in order to avoid the risk for laparoscopic conversion to open surgery [23,25,42]. Nephron-sparing partial nephrectomy is performed in the focal form, although the preoperative diagnosis is often not possible [45,46]. The etiology of XGP was related to stone disease in 83.3% of patients. The correct pre-operative diagnosis of XGP occurred in only 66.7% of patients with high stages of disease (II and III); in the stage I-nephric form, CT imaging could not differentiate XGP from other renal inflammatory disease [23], and our result underlines that the invasiveness of XGP in high stages of disease can support a confident diagnosis, but conversely, in the mild stage, only a nonspecific diagnosis of renal inflammatory disease can be formulated. On the other hand, our data reflect that patients with high-stage XGP are more common, possibly because in the low stage, the pathology is underestimated. Our study has several limitations: it is a retrospective, single-center study and the number of patients is relatively small.

## 5. Conclusions

Although XGP is considered a rare disease, at our institution, it represented 43.90% among benign nephrectomies. XGP is related to stone disease in 83.33% of cases. As described in other studies, we found a population that consisted largely of middle-aged women presenting with nonspecific symptoms and laboratory values. CT diagnostic accuracy for kidney inflammatory disease was extremely high, whereas the suspected diagnosis of XGP was formulated preoperatively in only 66.67% of high-stage diseases, where the hallmarks of invasiveness and fistulization of the pathology increased the diagnostic confidence. The stage I-nephric form of XGP could not be differentiated, based on imaging, from other renal inflammatory disease. The “bear paw “sign remains the more characteristic finding of CT (72.22%) in XGP; meanwhile, the presence of xanthomas, although considered pathognomic, cannot be reliable for the diagnosis because it is extremely rare (5.56%).

## Figures and Tables

**Table 1:** Descriptive analysis of XGP among all patients.

	XGP			*p*-Value
	NO (*n* = 23)	YES (*n* = 18)	TOTAL *n* = (41)	
**Demographic data**				
Age (years), mean (SD)	54 (20)	67 (11)	60 (17)	**0.011**
Sex (female), *n* (%)	13 (56.5)	13 (72.2)	26 (63.4)	0.300
**Symptoms**				
Flank pain, *n* (%)	15 (65.2)	13 (72.2)	28 (68.3)	0.742
Fever, *n* (%)	4 (17.4)	6 (33.3)	10 (24.4)	0.289
Palpable mass, *n* (%)	0 (0.0)	2 (11.1)	2 (4.5)	0.187
**Comorbidities**				
Diabetes, *n* (%)	9 (39.1)	13 (72.2)	22 (53.7)	**0.035**
Hypertension, *n* (%)	0 (0.0)	3 (16.7)	3 (7.3)	0.077
Hematuria, *n* (%)	6 (26.1)	3 (16.7)	9 (22.0)	0.706
**Laboratory analysis**				
Hemoglobin (g/dL), median (IQ–3Q)	12.6 (9.4–14.6)	9.6 (8.6–12.2)	10.9 (8.9–13.4)	**0.020**
Leucocytes (10^3^ mm^3^), median (IQ–3Q)	9.6 (5.5–12.2)	11.3 (9.1–16.2)	10.7 (7.13–12.9)	**0.033**
Cholinesterase (U/L), mean (SD)	6837 (2554)	4166 (2442)	5704 (2809)	**0.005**
Creatinine (md/dL), median (IQ–3Q)	1.2 (1.0–1.9)	1.4 (1.0–2.2)	1.3 (1.0–2.0)	0.627
Urea (mg/dL), median (IQ–3Q)	44 (31–61)	45 (38–87)	44 (33–62)	0.415
Procalcitonin (mg/dL), median (IQ–3Q)	7.1 (1.9–25.5)	16.7 (7.8–23.3)	14.6 (4.4–25.2)	0.304
**CT Findings**				
Staghorn calculi, *n* (%)	1 (4.4)	10 (55.6)	11 (26.8)	<0.001
Stone disease, *n* (%)	14 (70.9)	15 (83.3)	29 (70.7)	0.171
Bear paw sign, *n* (%)	0 (0.0)	13 (72.2)	13 (31.7)	**<0.001**
Xantoma, *n* (%)	0 (0.0)	1 (5.6)	1 (2.4)	0.439
Hydronephrosis, *n* (%)	21 (91.3)	18 (100.0)	39 (95.1)	0.495
Pyonephrosis, *n* (%)	5 (21.7)	16 (88.9)	21 (51.2)	**<0.001**
Stent, *n* (%)	4 (17.4)	7 (38.9)	11 (26.8)	0.164
Air in the collecting system, *n* (%)	1 (4.4)	6 (33.3)	7 (17.1)	**0.031**
Blood in the collecting system, *n* (%)	3 (13.0)	5 (27.8)	8 (19.5)	0.267
Fat stranding, *n* (%)	7 (30.4)	16 (88.9)	23 (56.1)	**<0.001**
Renal abscess, *n* (%)	4 (17.4)	11 (61.1)	15 (36.6)	**0.008**
Perirenal abscess, *n* (%)	4 (17.4)	13 (72.2)	17 (41.5)	**0.001**
Pararenal abscess, *n* (%)	3 (13.0)	10 (55.6)	13 (31.7)	**0.006**
Spleen abscess, *n* (%)	0 (0.0)	4 (22.2)	4 (9.8)	**0.030**
Psoas abscess, *n* (%)	2 (8.7)	9 (50.0)	11 (26.8)	**0.005**
Liver abscess, *n* (%)	0 (0.0)	2 (11.1)	2 (4.9)	0.187
Fistulization, *n* (%)	2 (8.7)	6 (33.3)	8 (19.5)	0.109
Pleural effusion, *n* (%)	4 (17.4)	10 (55.6)	14 (34.2)	**0.010**
Lung atelectasis/disventilation, *n* (%)	6 (26.1)	12 (66.7)	18 (43.9)	**0.009**

**Table 2:** Univariable analysis of XGP.

	Cases with Available Data		
		OR (95%CI)	*p*-Value
**Demographic data**			
Age > 55	41	12.4 (2.3–67.6)	**0.003**
Sex (female)	41	2.0 (0.5–7.5)	0.304
**Symptoms**			
Flank pain	41	1.4 (0.4–5.3)	0.633
Fever	41	2.4 (0.6–10.2)	0.245
Palpable mass	41	-	-
**Comorbidities**			
Diabetes	41	4.0 (1.1–15.3)	**0.039**
Hypertension	41	-	-
Hematuria	30	0.4 (0.1–1.9)	0.239
**Laboratory analysis**			
Hemoglobin < 11.5 g/dL	40	2.9 (0.8–10.6)	0.109
Leucocytes > 10.5 10^3^/ mm^3^	41	4.0 (1.1–15.3)	**0.039**
Cholinesterasis (<4000 U/L for female and <5000 U/L for male)	16	6.8 (1.4–31.9)	**0.016**
Creatinine > 0.95 md/dL	41	0.5 (0.1–2.4)	0.429
Urea > 50 mg/dL	41	1.5 (0.4–5.3)	0.530
Procalcitonin > 0.5 mg/dL	26	2.6 (0.2–32.9)	0.461
**CT Findings**			
Staghorn calculi	41	27.5 (3.0–250.5)	**0.003**
Lithiasis	41	3.2 (0.7–14.3)	0.126
Hydronephrosis	41	-	-
Bear paw sign	41	-	-
Xantoma	41	-	-
Pyonephrosis	41	28.8 (4.9–169.5)	**<0.001**
Stent	41	3.0 (0.7–12.7)	0.131
Air in the collecting system	41	11.0 (1.2–102.4)	**0.035**
Blood in the collecting system	41	2.6 (0.5–12.6)	0.247
Fat stranding	41	18.3 (3.3–101.9)	**0.001**
Renal abscess	41	7.5 (1.8–31.4)	**0.006**
Perirenal abscess	41	12.4 (2.8–54.9)	**0.001**
Pararenal abscess	41	8.7 (1.8–38.4)	**0.003**
Spleen abscess	41	-	-
Psoas abscess	41	10.5 (1.9–58.6)	**0.007**
Liver abscess	41	-	-
Fistulization	41	5.3 (0.9–30.2)	0.063
Pleural effusion	41	5.9 (1.4–24.7)	**0.014**
Lung atelectasis/disventilation	41	5.7 (1.5–21.9)	**0.012**

**Table 3:** Multivariate analysis of XGP.

	OR (95%CI)	*p*-Value
**Age**		
≤55	Reference	
>55 years	41.5 (1.8–979.3)	**0.021**
**Staghorn calculi**		
No	Reference	
Yes	61.8 (1.5–2496.2)	**0.029**
**Pyonephrosis**		
No	Reference	
Yes	19.0 (0.99–364.8)	0.051
**Pleural effusion**		
No	Reference	
Yes	10.3 (0.5–206.9)	0.129

**Table 4:** CT findings in stages of XGP.

	Stage I (*n* = 5)	Stage II (*n* = 2)	Stage III (*n* = 11)	Total (*n* = 18)	*p*-Value
**Staghorn calculi, *n* (%)**	2 (40.0)	2 (100.0)	6 (54.5)	10 (55.6)	0.638
**Stone disease, *n* (%)**	4 (80.0)	2 (100.0)	9 (81.8)	15 (82.3)	0.999
**Hydronephrosis, *n* (%)**	5 (100.0)	2 (100.0)	11 (100.0)	18 (100.0)	-
**Bear paw sign, *n* (%)**	3 (60.0)	2 (100.0)	8 (72.7)	13 (72.2)	0.999
**Xantoma, *n* (%)**	1 (20.0)	0 (0.0)	0 (0.0)	1 (5.6)	-
**Pyonephrosis, *n* (%)**	3 (60.0)	2 (100.0)	11 (100.0)	16 (88.9)	0.137
**Stent, *n* (%)**	2 (40.0)	1 (50.0)	4 (36.4)	7 (38.9)	0.999
**Air in the collecting system, *n* (%)**	1 (20.0)	1 (50.0)	4 (36.4)	6 (33.3)	0.999
**Blood in the collecting system, *n* (%)**	1 (20.0)	1 (50.0)	3 (27.3)	5 (27.8)	0.999
**Fat stranding, *n* (%)**	3 (60.0)	2 (100.0)	11 (100.0)	16 (88.9)	0.137
**Renal abscess, *n* (%)**	0 (0.0)	1 (50.0)	10 (90.9)	11 (61.1)	0.001
**Perirenal abscess, *n* (%)**	0 (0.0)	2 (100.0)	11 (100.0)	13 (72.2)	<0.001
**Pararenal abscess, *n* (%)**	0 (0.0)	0 (0.0)	10 (90.9)	10 (55.6)	<0.001
**Extrarenal abscess, *n* (%)**	0 (0.0)	0 (0.0)	10 (90.9)	10 (55.6)	<0.001
**Fistulization, *n* (%)**	0 (0.0)	0 (0.0)	6 (54.5)	6 (33.3)	0.070
**Pleural effusion, *n* (%)**	2 (40.0)	1 (50.0)	7 (63.6)	10 (55.6)	0.789
**Lung atelectasis/disventilation, *n* (%)**	2 (40.0)	1 (50.0)	9 (81.8)	12 (66.7)	0.253

## Data Availability

Data are available on request.
